# Innovative Nutrition Education: A Color-Coded Tool for Individuals with Low Literacy Level

**DOI:** 10.3390/healthcare10020272

**Published:** 2022-01-30

**Authors:** Hiba Bawadi, Ghadir Fakhri Al-Jayyousi, Hala Shabana, Sana Boutefnouchet, Sereen Eljazzar, Shrooq Ismail

**Affiliations:** 1 Department of Human Nutrition, College of Health Sciences, QU Health, Qatar University, Doha P.O. Box 2713, Qatar; hs1205949@student.qu.edu.qa (H.S.); sb1204296@student.qu.edu.qa (S.B.); se1203396@student.qu.edu.qa (S.E.); si1108542@student.qu.edu.qa (S.I.); 2 Department of Public Health, College of Health Sciences, QU Health, Qatar University, Doha P.O. Box 2713, Qatar; g.aljayyousi@qu.edu.qa

**Keywords:** exchange list system, low literacy, meal planning, workers, Qatar

## Abstract

(1) Background: The food exchange system was developed to serve as an educational tool in helping individuals plan their own meals. This study aimed to develop a friendly-user food exchange list for individuals with a low literacy level; (2) Methods: A two-group, pre-test/post-test research study aimed to develop a friendly-user food exchange list for individuals with a low literacy level. Thirty female workers of low literacy were recruited. Participants were divided into two groups. Group one was taught how to use the standard exchange system, while group two was taught how to use the modified exchange system. Each participant was assigned a task of prepare a meal with a specified caloric content and macronutrient distribution. The task was assigned before and after the exchange list education session. Groups’ differences were tested using the chi-square test, and the analysis of variance (ANOVA); (3) Results: A higher percentage of participants in group two were able to plan daily diets that achieved the recommendations of fruits (*p* = 0.02), protein (*p* = 0.03), dairy (*p* < 0.001), carbohydrates (*p* < 0.001), and calories (*p* < 0.001). Moreover, diet plans prepared by group two had a higher healthy eating index (*p* < 0.001) when compared to diet plans prepared by group one. The modified exchange lists are a friendly-user tool that can be implemented for individuals with low literacy, since it relies on visual techniques.

## 1. Introduction

Also known as chronic diseases, non-communicable diseases (NCDs), including cardiovascular diseases (CVDs), diabetes, and cancer, affect people in the long term [1]. The World Health Organization (WHO) reported a growing number of deaths from NCDs worldwide [2]. A healthy, balanced diet has shown to reduce the risk of NCDs [3].

The food exchange system was developed to serve as an educational tool helping individuals plan their own meals. It is based on the nutritional value of different items, which can be interchanged with one another to offer a wide variety of choices in the diet [4]. The system was primarily designed to assist diabetic patients to manage their carbohydrate intake. The conventional exchange system divides the amounts of carbohydrates between meals, and patients should plan their meals according to the quantities of carbohydrates they are allowed to consume. The system is as useful as the standard meal planning approach, because it provides users with a wide variety of choices from which they can plan their own healthy and balanced diet [5]. 

Low-income populations often face difficulties with food costs and have limited options for healthy food selection. A similar concept applies to social minorities. Meal plans with limited choices of foods could discriminate against cultural differences when planning for minorities’ diets [6]. The use of food exchange systems can solve such issues. Involving users with the selection of their own food allows them to plan for healthy meals that meet their budget, as well as personal and cultural preferences [7,8,9]. A basic understanding of nutrition and basic reading, writing, and math skills at the high-school level at the least are essential for using this list [10]. In developing countries, people may not have proper access to education, which may influence their nutrition status because of their reading ability and low literacy [11]. Patients with low literacy struggle to manage NCDs and general self-care [12]. To overcome the literacy barrier, the Center for Disease Control and Prevention (CDC) developed a guide called “Simply put: A guide for creating easy-to-understand materials” [13]. The booklet aims to help professionals translate complex scientific information into information that is easy to understand and use by low-literacy individuals. The guide provides steps to create easy-to-understand materials supported by evidence-based data. 

In Qatar, food habits have shifted from a healthy pattern to an unhealthy, Westernized diet [14], associated with an increased risk of NCDs and high mortality rates [15]. Qatar relies heavily on migrant workers for the bulk of its workforce. The migrants working in Qatar come from diverse ethnic backgrounds and they comprise 86% of the population and 94% of the workforce [16]. Migrant workers are more vulnerable to healthcare inequities and are at risk from disparities in their access to healthcare services for several reasons, including income and education [17,18]. The healthcare system suffers from a gap between individuals’ literacy states and the literacy level at which nutritional interventions are provided. This study aimed to develop a friendly-user modified exchange list and to evaluate its effectiveness regarding meal planning skills among workers with low literacy levels.

## 2. Materials and Methods

**Figure 1:**
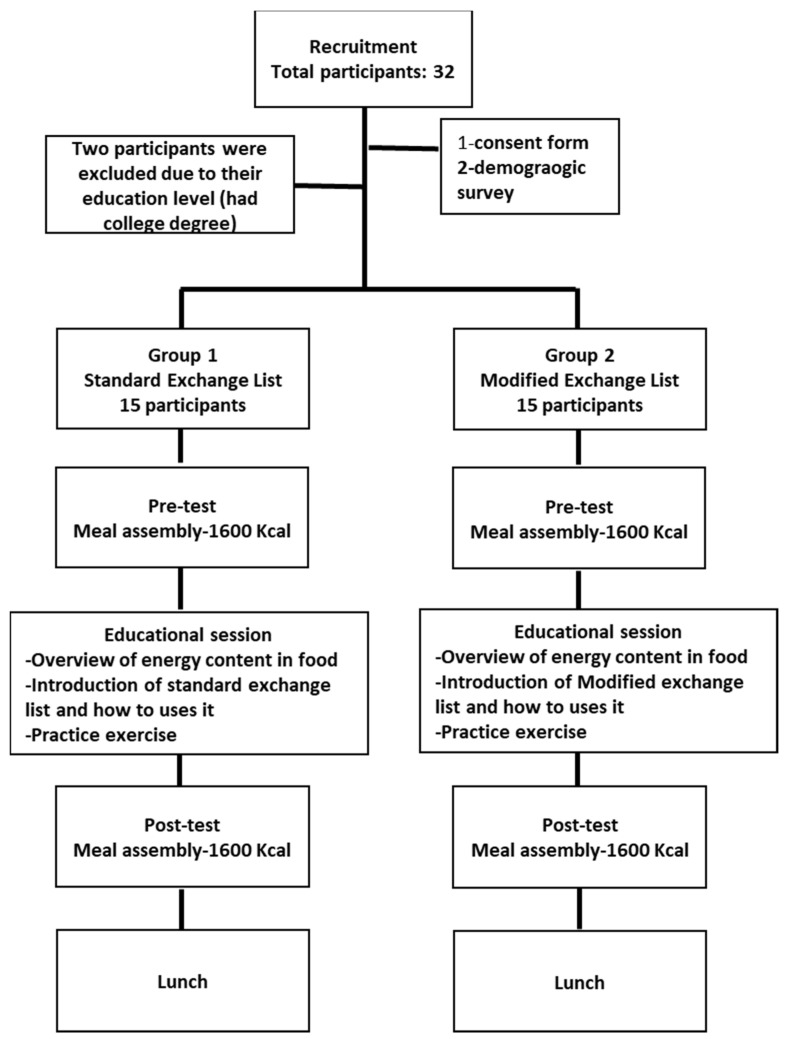
Study flow.

This was a two-group, pre-test/post-test study to assess the efficacy of a modified exchange list against the standard one. This project was conducted at Qatar University (QU) for a total period of two weeks. The housekeeping supervisor chose a convenient sample of workers to participate in our study. The participants were randomly assigned to either the standard or modified group. This research was approved by the Institutional Review Board at QU. Figure 1 presents a flowchart of the study. 

### 2.1. Study Participants

A list of potential participants was obtained from the housekeeping department at QU. The convenient sampling strategy was employed to recruit workers based on their work schedule. Based on the poverty line of QAR 3514 [19], the income for housekeeping employees (500–1500 QR), this is considered a low income in the State of Qatar. All housekeeping employees recruited for this study had only an education level of high school or less. In addition to low education (less than 12 years of formal education) and income levels (below poverty line). An interview-based demographic questionnaire was completed by the participants that included questions on their age, nationality, gender, education level, average family income, and whether they suffered from any diseases. 

The study outcome variable (meal-planning skills) was measured by a one-day meal plan test using food models. The quality of the participants’ meal plan was assessed by calculating the healthy eating index (HEI) [20] of the meal plan they would create.

The pre-intervention assessment included showing the participants 145 food models (Numed, Lebanon) and giving them a sheet to demonstrate a 1600 kcal diet. After that, they were asked to prepare a one-day diet plan using the food models provided. The participants were asked to make meals for breakfast, lunch, and dinner; they also had the option of creating one to two snacks. The meal-planning test was conducted in a separate room where only one participant was allowed to enter the room and perform the meal-plan test. Because the purpose of the meal planning was for testing participants’ skills and not for food consumption, the choice of the calorie content of the test was fully based on each participant’s physical characteristics (weight, height, physical activity), rather than a common diet calorie content of 1600 being chosen. Photographs of the meal plans were captured and coded based on the participants’ assigned identifications. Meal plans were entered into Super Tracker [21] to analyze the nutrient content and food group distribution. To meet the targets of calories (±5%), the levels of fruits, vegetables, dairy, carbohydrates, protein, and fat were recorded for each participant. 

The HEI for the meal plan was also measured as an indicator of the meal plan’s quality prepared by the study’s participants. The HEI had been established through the United States Department of Agriculture’s (USDA) Center for Nutrition Policy and Promotion to evaluate how well Americans’ diets conform to nutritional guidelines [22]. The HEI managed to integrate nutrient needs and nutritional guidelines into a single measure. Furthermore, this index was used to indicate a 12-component system comprising five nutritional groups and a measure of diversity in diet consumption. A scoring system starting from 0 to 10 was used to assess each component of the HEI, which indicated an overall HEI score of 100 [22].

### 2.2. Intervention

Participants in the treatment group (modified) received a group nutritional training session based on an innovative presentation of the exchange lists that are user-friendly, free of technical terms, and color-coded. Participants in the control group received a group nutritional training session based on the standard exchange lists. To account for the participants’ low literacy level in both groups, the training session was prepared following the CDC regulations for the Simply Put guide [13]. Content of the presentations focused mainly on the most important details the participants needed to know, including the concept of a food exchange system and how to use it. Extensive text use was limited; instead, more visuals were used to attract the participants’ attention and facilitate understanding the concepts. The participants were also provided with instructions and steps on developing a meal plan using an exchange system. In addition, they were asked to interact with the provided material by completing a task involving developing a meal plan, with educators’ assistance [13].

#### 2.2.1. Meal-Planning Training Using Standard Exchange Lists

**Figure 2:**
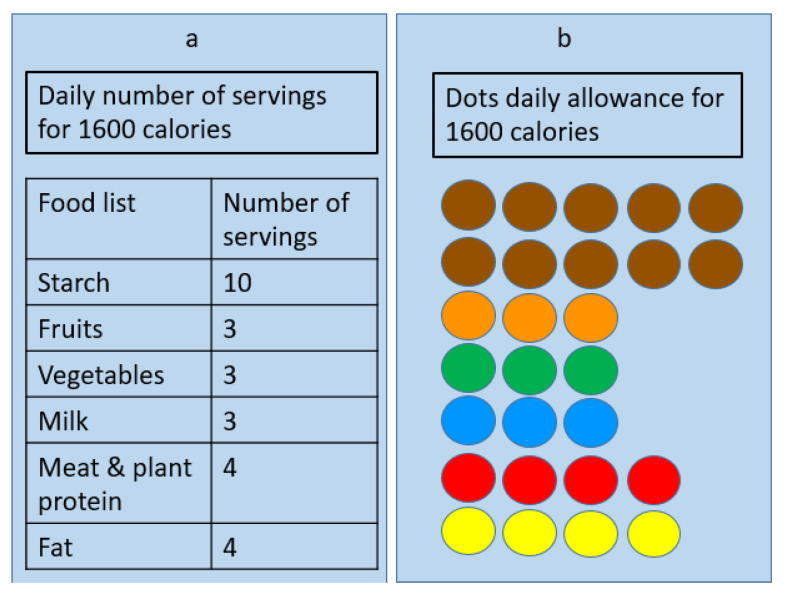
One-day diet plan to achieve 1600 calories. (**a**) Presents the recommended number of servings from participants in group and (**b**) presents the color-coded dots participants needed to assemble by matching the equivalent dots attached to each food model.

The training session for the standard exchange lists mainly focused on the concept of serving sizes and their translation into portion sizes. The session revolved around grouping bases of foods and explaining the concept of “serving” and serving size. The session also described how to estimate intake based on the allowed servings within a day. Participants were given a certain number of servings per food group and a daily caloric allowance (Figure 2a). Food models were labeled in exchange formats, with a sticker on an apple model, for example, labeled as one exchange of fruit.

#### 2.2.2. Meal-Planning Training Using Modified Exchange Lists

The training session for the modified exchange lists mainly focused on selecting food items based on the corresponding color associated with its group. The concept of the food exchange system was introduced. Participants in the modified exchange list were given a sheet of paper with color-coded dots, where each dot represented one serving from each group. For example, instead of mentioning the starch group (likewise in the standard exchange lists), a brown dot was assigned for each serving of starch. All food models belonging to the starch group had a sticker with a brown dot on them. Participants selected the food item, checked the dots equivalency on the attached sticker and then—upon selection—cross out these dots from the dot allowance provided (Figure 2b).

It is important to point out that for each diet plan, there should be pre-determined dots depending on the caloric content. The dots, equivalent to 1600 Kcal, were provided to the participants in our study, and for any diet plan with specific caloric content, the equivalent dots must be provided. Thus, if the choice was 1800 Kcal, a number of dots equaling 1800 Kcal should have been provided, etc.

#### 2.2.3. Post-Test

After the training session, the participants were asked to prepare a one-day meal using the food models. The participants were asked to look at all the food models and were given the same sheet of paper, depending on which exchange list they were working with, containing 1600 calories for the day. Then, they were asked to prepare three meals and one or two snacks. Time was recorded for all the participants, and photographs were taken for each meal. For the standard exchange list, food models were labeled with their corresponding serving sizes and food categories. For the modified exchange list, food models were relabeled with their corresponding colored dots to represent food groups. After taking the post-test, the participants were given a healthy lunch.

### 2.3. Statistical Analysis

Data were analyzed by the statistical package of social sciences (SPSS). Mean (M), and standard deviation (SD) were performed for continuous variables such as age. Descriptive statistics including frequencies (n) and percentages were computed to examine categorical variables. The distribution of group differences was tested using the chi-square test, and the differences in the HEI were tested using the analysis of variance (ANOVA). The results were controlled for age and gender. Results with *p*-values < 0.05 were considered to be statistically significant.

## 3. Results

A total of 32 female workers at QU agreed to participate in both meetings (Table 1). The study included only 30 female workers with low literacy. The participants were divided into two groups of 15 participants in the standard and modified groups. The participants’ mean age within the standard exchange list group was 31.1 years old, with a standard deviation of ±1.6. The mean age within the modified exchange list group was 29.9 years old, with a standard deviation of ±1.1. Within the standard group, 66.7% of the participants had a high school degree, while 66.7% of the participants in the modified group had a primary degree.

For the standard exchange list group, most of the participants (66.7%) had an income ranging between 500 and 999 Qatari Riyal (QR) per month. For the modified exchange list, the majority (86.7%) had an income ranging between 500 and 999 QR per month. Approximately 73% of the participants from the standard exchange list group were healthy, while the rest suffered from health problems (e.g., diabetes). The majority of the participants from the modified exchange list were also healthy, except for 33.3% who suffered from health problems, such as diabetes.

Table 2 describes the food groups and the nutrient content of the meal plans prepared by the participants in the standard and modified groups before and after the training session. Food types were divided into seven categories as per the American Dietary Guidelines’ classification of food items. The last three rows represent the Acceptable Macronutrient Distribution Range (AMDR) values, as recommended by the Academy of Nutrition and Dietetics. The table provides information on the number and percentage of the participants who met their daily dietary needs from each food category within each group.

With regards to whole grains content in the meal plan, no difference was noticed between the two groups for both the pre-test and post-test. No significant difference was observed between the two groups for refined grains content. During the pre-test, the planned diets of 6.6% of the participants from the standard group and 13.3% from the modified group met the daily refined grains target (*p* = 0.5). In the post-test, the planned diets of 6.6% of the participants from the standard group and 26.6% of the participants from the modified group met their refined grains’ intake requirement (*p* = 0.16).

Fruit content in the diet plans was also studied. In the pre-test, the planned diets of 60% of the participants from the standard group and 86% of the participants from the modified group met the recommendations for fruits (*p* = 0.1). During the post-test, all the participants of the modified group met their daily intake requirements for fruits. However, only 66% in the standard group met their fruit intake requirement for the day. The difference between the two groups was statistically significant (*p* = 0.02). With regards to the vegetable content in the prepared diet plans, a higher percentage of diet plans prepared by the modified groups met the recommendations for vegetables (*p* = 0.10).

For the dairy product content in the diet plans, only 6.6% from the standard group and 13.3% from the modified group met their daily intake requirements for dairy food in the pre-test (*p* = 0.50). For the post-test, none of the participants from the standard group met their daily requirements for dairy, while 73.3% of the participants from the modified group met their requirements. The difference was noted to be significant between the two groups during the post-test (*p* ˂ 0.001).

As for protein content, 33.3% of the participants from the standard and 46.7% from the modified group met their daily intake requirements for protein (*p* = 0.35). During the post-test, the percentage of the participants from the standard group who met their protein requirements decreased to 26.7%. On the contrary, the percentage of the participants from the modified group who met their protein requirements reached 66.7% in the post-test. The difference between the two groups during the post-test was found to be significant (*p* = 0.03).

Regarding the saturated fat content in the diet plans, 73.3% of the participants from the standard group and 66.6% of the participants from the modified group met their saturated fat intake requirement for the day in the pre-test (*p* = 0.5). During the post-test, 66.6% of the participants from the standard group and 80% of the participants from the modified group met their saturated fat intake requirements for the day (*p* = 0.16).

Concerning meeting the caloric goal of 1600 kcal (±5%), only 13.3% of the participants in both the standard and modified groups met their daily energy requirements in the pre-test (*p* = 0.70). In the post-test, the percentage of participants who met their daily energy requirement decreased to 6.6% in the standard group and reached 100% in the modified group. The difference between the two groups during the post-test was found to be significant (*p* ˂ 0.001).

During the pre-test, the percentage of diet plans prepared by the participants from the standard group that met the daily AMDR for carbohydrates was 46.6%, compared to 60% for diet plans prepared by the modified group (*p* ˃ 0.5). Similar results were found regarding protein and fat AMDR. In the post-test, the percentage of diet plans prepared by the participants from the standard group that met the daily AMDR for carbohydrates was 46.6%, compared to 80% for diet plans prepared by the modified group (*p* ˂ 0.001). The percentage of diet plans prepared by the participants from the standard group that met daily AMDR for fat was 60%, compared to 80% for diets planned by the modified group, albeit no significant difference was detected (*p* = 0.21). All participants (100%) from both groups met their daily AMDR requirements for protein during the post-test.

**Figure 3:**
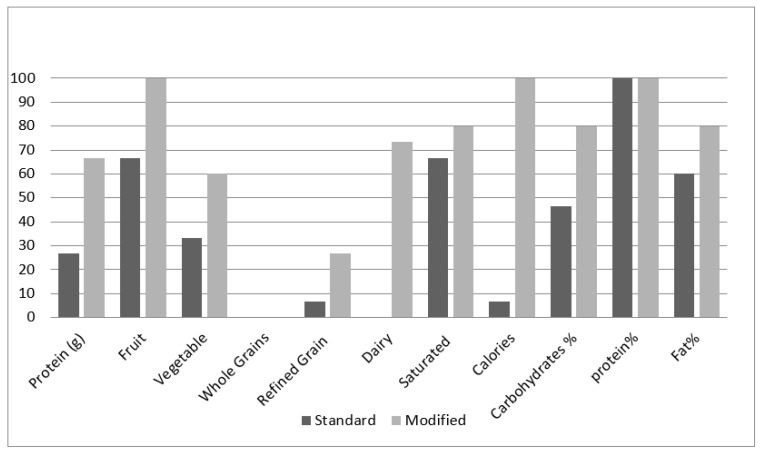
Number of participants in both research groups who achieved the nutritional recommendations in their planned diet plans.

Figure 3 shows the differences between the health index (HEI) scores for the standard and modified groups for the pre-and post-test. In the standard group, the HEI mean score slightly increased from 62.6% in the pre-test to 67% in the post-test. The HEI for the modified group increased from 70.8% in the pre-test to 86% in the post-test. The comparison of results between the standard and modified groups showed no statistical significance in the pre-test (*p* = 0.058) when compared to the statistical significance (*p* = 0.00) in the post-test.

## 4. Discussion

In this educational intervention study for low-literacy individuals, the simplified color-exchange list improved the participants’ meal-planning skills. A modified exchange list is an easy tool to help patients with or without diabetes in meal planning. Overall, the participants achieved better results in the modified exchange list than those in the standard exchange list. Participants in the modified exchange group planned a much healthier and nutritionally rich meal. This increased their knowledge of and skill in planning their own meals despite their limited education or low literacy level.

The total post-test HEI mean score of the participants who had received the modified exchange list training session was 86%, while those in the standard exchange list training session had a mean of 67%. The HEI scoring scale (Table 2) indicates that a total score of >80% is classified as a “good” diet while having a total score of 51–80% is classified as a diet that “needs improvement.” This is consistent with our findings, as the modified exchange list post-test HEI total score suggests that the participants’ diet is good, while the standard exchange list post-test HEI total score indicates that their diet needs improvement.

Another means of comparison is by assessing the nutrients and the AMDR in both groups and comparing them against the Qatari and American dairy guideline recommendations. Table 2 shows no significant difference in the pre-test in both groups. A significant difference was noticed in the post-test for fruits, protein, dairy consumption, total caloric diet plan, and carbohydrate percentage, where the modified exchange list group had higher scores. This supports the fact that the modified color-coded exchange list can improve the knowledge and understanding of low-literacy individuals.

A cross-sectional study showed that low-literacy individuals may struggle to understand food and drug labels due to their complexity [22]. This supports our study’s objective in using and modifying tools or labels aimed to educate public consumers from various educational levels. Wolff and his colleagues [23] state that many diabetic patients may not be able to obtain maximum benefits from the available educational tools made for people with diabetes, as they require advanced reading and writing levels without accounting for patients with low literacy. Our study results endorse that statement by showing the significant differences between the two groups’ HEI scores (*p* < 0.001). They [23] also mention that diabetes management programs addressing health literacy might be associated with improved glycemic control in low-literacy patients. This illustrates the importance of targeting groups with a low education level and trying to use colors or pictures to advance their understanding of health and nutrition.

Studies show that when patients are given simple, direct, and concise instructions, they are better able to follow them than when given general information [24]. The modified group’s results improved more quickly than those of the standard group, as evidenced by the HEI (*p* ≤ 0.001) for the modified group and (*p* = 0.58) for the standard group. An example given is to prescribe patients to take a tablet “every 12 h” instead of “twice a day”, to avoid any doubt [24]. Many people with limited health literacy are unable to follow simple prescription directions if left open ended [24]. Prescriptions should be as specific as possible. This explains why participants achieved great results in the modified exchange list group, because they were given interactive examples, which aided their understanding of the topic.

The modified exchange list was developed to simplify food requirements for individuals. The modified exchange list has simple information and requires little background information; it is all about colors and matching dots. The way participants are given the colored dots facilitates the easiest possible way of creating a healthy meal. The standard exchange list, however, is very strict and full of words described by serving sizes, measurements, and numbers. Studies show that instructions are applicable when shown in an attractive layout [2]. The use of food models as a demonstration gave learners a hands-on experience of how to plan a meal. When comparing the test sheets given to the participants, the modified exchange list did not provide details on the types of food or the number of calories. Instead, it created a visual depiction of the serving sizes.

The modified exchange list can be universal, with each color representing a type of food group. Therefore, it does not need further translation once the individual understands that. As is evident in the results, all the participants in the modified list achieved their calorie requirements in the post-test. A plan with no number or food category and lacking technical terms allows no room for misunderstanding. Our study suggests an improvement in health and self-care among low-income patients who receive visual tools. The findings of this study support the need for more innovative educational tools that includes fewer technical terminologies, which may form a huge barrier against implementation.

## 5. Limitations

This study has a few limitations. First, the study’s small sample size did not allow the researchers to draw conclusive results about the effectiveness of using the modified exchange system among the low-literacy populations. Therefore, further studies should test the use of the modified exchange list on a larger scale. Second, the absence of follow-up research limited the study’s ability to test the modified exchange system’s long-term effects on commitment among low-literacy individuals. Third, the study’s outcomes reflected meal-planning knowledge and skills. However, they failed to reflect the real-life application of the requisite skills. Changes in body weight or biochemical indicators may better reflect the effectiveness of the modified food exchange lists. Finally, we have included only women participants in our study. Our findings are not gender-specific, thus future studies should also include men.

### Implications for Research and Practice

Dietitians and health educators can work together to integrate the modified exchange lists into nutrition interventions for diabetic and non-diabetic individuals with a view to improving their overall diet. Future research is needed to monitor the impact of understanding the exchange lists on daily eating habits and measure the clinical outcomes for diabetic patients.

## 6. Conclusions

Understanding the needs and characteristics of a target audience is a crucial step to developing appropriate materials. Identifying literacy levels and the interests of a target audience allows professionals to use training sessions wisely. It also allows them to provide more specific and focused material. Our study supports the view that the modified exchange list provides an easier and more effective educational tool in planning healthy meals among individuals with low literacy. Simplifying the dietary instructions in any intervention is critical for success. The findings promise to improve the health of individuals with a low literacy level and socioeconomic status.

## Figures and Tables

**Table 1:** Sample characteristics for participants in modified & standard group (*n* = 30).

	Standard	Modified	*p*-Value
Age *	31.1 ± 1.6	29.9 ± 1.1	0.4 †
Education ‡			
Primary	3 (20)	7	0.09 §
Middle	2 (13.3)	4	
High	10 (66.7)	4	
Income ‡			
500–999	10	13	0.2 §
1000–1499	5	2	
Disease Status ‡			
Diseased	4	5	0.7 §
Not Diseased	11	10	

* Values presented as mean ± standard error of the mean. † *p*-value obtained from ANOVA. ‡ values presented as frequency (percentage). § *p*-values obtained from Chi-square test.

**Table 2:** Nutrient composition among standard and modified exchange list groups in the pre-and post-test.

Dietary Recommendations ^†^	Pre-Intervention		*p*-Value	Post Intervention		*p*-Value
	Standard	Modified		Standard	Modified	
Frui t ≥ 1.6 cup	9 (60)	13 (86.6)	0.1	10 (66.6)	15 (100)	0.02
Protein ≥ 4.4 oz	5 (33.3)	7 (46.7)	0.355	4 (26.7)	10 (66.7)	0.033
Whole Grain > 2.4 oz	0 (0)	0 (0)	__	0 (0)	0 (0)	__
Refined Grain < 2.4 oz	1 (6.6)	2 (13.3)	0.5	1 (6.6)	4 (26.6)	0.165
Vegetables ≥ 2 cup	4 (26.6)	3 (20)	0.5	5 (33.3)	9 (60)	0.1
Dairy ≥ 2.4 cup	1 (6.6)	2 (13.3)	0.5	0 (0)	11 (73.3)	<0.001
Saturated Fat < 10%	11 (73.3)	10 (66.6)	0.5	10 (66.6)	12 (80)	0.3
Total Calories 1520–1680 kcal	2 (13.3)	2 (13.3)	0.701	1 (6.6)	15 (100)	<0.001
CHO 45–65%	7 (46.6)	9 (60)	0.5	7 (46.6)	12 (80)	<0.001
Protein V	15 (100)	14 (93.3)	0.5	15 (100)	15 (100)	__
Fat 20–35%	7 (46.6)	10 (66.6)	0.231	9 (60)	12 (80)	0.213

Values are the number of participants in each research group (*n* = 1 who were able to meet their dietary recommendations (percentage) in the pre- and post-tests. ^†^ *p*-value obtained from ANOVA.

## Data Availability

The data presented in this study are available on request from the corresponding author.
